# Antihyperglycemic Effect of *Lavandula pedunculata*: In Vivo, In Vitro and Ex Vivo Approaches

**DOI:** 10.3390/pharmaceutics13122019

**Published:** 2021-11-26

**Authors:** Salima Boutahiri, Mohamed Bouhrim, Chayma Abidi, Hamza Mechchate, Ali S. Alqahtani, Omar M. Noman, Ferdinand Kouoh Elombo, Bernard Gressier, Sevser Sahpaz, Mohamed Bnouham, Jehan-François Desjeux, Touriya Zair, Bruno Eto

**Affiliations:** 1 Laboratoires TBC, Laboratory of Pharmacology, Pharmacokinetics and Clinical Pharmacy, Faculty of Pharmacy, University of Lille, F-59000 Lille, France; boutahirisalima@gmail.com (S.B.); ferdinand.kouohelombo@univ-lille.fr (F.K.E.); gressier.bernard@univ-lille.fr (B.G.); eto.bruno@univ-lille.fr (B.E.); 2 Research Team of Chemistry of Bioactive Molecules and the Environment, Laboratory of Innovative Materials and Biotechnology of Natural Resources, Faculty of Sciences, Moulay Ismaïl University, B.P. 11201 Zitoune, Meknes 50070, Morocco; zair.touriya@umi.ac.ma; 3 Univ. Lille, University of Liège, University of Picardie Jules Verne, JUNIA, UMRT 1158 BioEcoAgro, Specialized Metabolites of Plant Origin, F-59000 Lille, France; sahpaz.sevser@univ-lille.fr; 4 Laboratory of Bioresources, Biotechnology, Ethnopharmacology and Health, Faculty of Sciences, Mohammed First University, B.P. 717, Oujda 60000, Morocco; bnouham.mohamed@ump.ac.ma; 5 Laboratory of Functional Physiology and Valorization of Bio-Resources, Higher Institute of Biotechnology of Beja, University of Jendouba, B.P. 382, Beja 9000, Tunisia; abidichayma07@gmail.com; 6 Laboratory of Biotechnology, Environment, Agri-Food, and Health, Faculty of Sciences Dhar El Mahraz, University Sidi Mohamed Ben Abdellah, P.O. Box 1796, Fez 30000, Morocco; 7 Department of Pharmacognosy, College of Pharmacy, King Saud University, Riyadh 11451, Saudi Arabia; alalqahtani@ksu.edu.sa (A.S.A.); onoman@ksu.edu.sa (O.M.N.); 8 Laboratory de Pharmacology and Toxicology (LPT), Unit of Aromatic and Medicinal Plants Valorization, Department of Biochemistry, Faculty of Sciences, University of Yaoundé 1, Yaoundé BP 812, Cameroon; 9 National Academy of Medicine, 16, rue Bonaparte, 75006 Paris, France; jehanfrancois.desjeux@gmail.com

**Keywords:** *Lavandula pedunculata* (Mill.) Cav., *Punica granatum* L., *Trigonella foenum-graecum* L., diabetes, glucose intestinal absorption, oral glucose tolerance test

## Abstract

*Lavandula pedunculata* (Mill.) Cav. (LP) is one of lavender species traditionally used in Morocco to prevent or cure diabetes, alone or in the form of polyherbal preparations (PHP). Therefore, the primary objective of this study was to test the antihyperglycemic effect of the aqueous extract of LP, alone and in combination with *Punica granatum* L. (PG) and *Trigonella foenum-graecum* L. (FGK). The secondary objective was to explore some mechanisms of action on the digestive functions. The antihyperglycemic effect of the aqueous extract of LP, alone and in combination with PG and FGK, was studied in vivo using an oral glucose tolerance test (OGTT). In addition, LP extract was tested on the activities of some digestive enzymes (pancreatic α-amylase and intestinal α-glucosidase) in vitro and on the intestinal absorption of glucose ex vivo using a short-circuit current (I_sc_) technique. Acute and chronic oral administration of LP aqueous extract reduced the peak of the glucose concentration (30 min, *p* < 0.01) and the area under the curve (AUC, *p* < 0.01). The effect of LP + PG was at the same amplitude to that of the positive control Metformin (MET). LP aqueous extract inhibited the pancreatic α-amylase with an IC_50_ almost identical to acarbose (0.44 ± 0.05 mg/mL and 0.36 ± 0.02 mg/mL, respectively), as well as the intestinal α-glucosidase, (IC_50_ = 131 ± 20 µg/mL) and the intestinal glucose absorption (IC_50_ = 81.28 ± 4.01 µg/mL) in concentration-dependent manners. LP aqueous extract exhibited potent actions on hyperglycemia, with an inhibition on digestive enzymes and glucose absorption. In addition, the combination with PG and FGK enhanced oral glucose tolerance in rats. These findings back up the traditional use of LP in type 2 diabetes treatment and the effectiveness of the alternative and combinative poly-phytotherapy (ACPP).

## 1. Introduction

Diabetes is a group of metabolic diseases of which blood hyperglycaemia is the main characteristic. This hyperglycaemia results from a lack of insulin secretion and/or a failure in insulin action. Type 1 diabetes is mostly induced by autoimmune diseases causing the destruction of the pancreatic β-cells responsible of the insulin production. In the more common type 2 diabetes, the main mechanism is insulin resistance. The latter is related to abnormalities in the insulin action on the target tissues (decrease in tissue responses and/or in insulin secretion) [1,2]. The long-term exposure to hyperglycaemia causes serious damages in the eyes (retinopathy), kidneys (nephropathy), and nerves (peripheral and autonomic neuropathy), as well as in the heart and blood vessels (cardiovascular and cerebrovascular diseases) [1]. 

In 2019, more than one million of the population under the age of 20 (children and teenagers) were living with type 1 diabetes. Moreover, 463 million people aged from 20 to 79 years, and representing 9.3% of adults, were living with diabetes. By this rate, the number of adults with diabetes is estimated to reach 578 million by 2030, and 700 million by 2045. The increasing prevalence of diabetes is due to an upsurge in type 2 diabetes. Changing lifestyle habits, like unhealthy diets, obesity, and sedentary lifestyles, are the main contributory factors leading to this increase. However, levels of childhood-onset type 1 diabetes are also on the rise [2].

Diabetes management requires the following of specific diets and physical exercises, as well as the use of medicines that regulate the blood sugar level. Individuals with type 1 diabetes (5–10% of the diabetic population) are dependent on insulin intake for survival. Type 2 diabetes patients (90–95% of the diabetic population) are treated with a variety of drugs with different modes of action. Among these drugs, biguanides (MET and phenformin) and thiazolidinediones (rosiglitazone and pioglitazone) decrease insulin resistance, and the α-glucosidase inhibitors (miglitol and acarbose) decrease glucose absorption from intestine. There are also other drugs like meglitinides which are insulin secretagogues, sulfonylureas that are blockers of the ATP sensitive potassium channels and glycosurics that inhibit reabsorption of glucose in the kidney. As for peptide analogs, gastric inhibitory peptide analogs glucagon like peptide-1 (GLP-1), injectable incretin mimetics, and injectable Amylin analogues, they increase incretin levels, which increases insulin secretion and inhibits glucagon release [3,4,5]. These conventional drugs may cause moderate to severe side effects, that is why research for alternative treatments is needed [6].

Medicinal plants are used across the world for their antidiabetic activity [7,8]. In Morocco, lavender is largely cited in different ethnobotanical and ethnopharmacological studies for its antidiabetic activity [9,10,11]. Moreover, it is often used by the population in the treatment of various diseases. Furthermore, researchers have been investigating the different activities of lavender and they were able to prove that it has antidiabetic, antimicrobial, anti-inflammatory, antirheumatic, antioxidant, antispasmodic, and antidepressant properties [12,13,14,15,16,17,18]. However, lavender is usually studied for the properties of its essential oils, regardless the large use of its aqueous extracts in traditional medicine [19,20]. 

According to Ghourri et al. (2013), medicinal plants are used essentially in the form of PHP to treat diabetes in the Moroccan Sahara (Tan-Tan). *Trigonella foenum-graecum*, *Nigella sativa*, *Lavandula stoechas, Origanum species*, *Rosmarinus officinalis,* and *Ammodaucus leucotrichus* are among the most used plants [21]. It was also reported that the antidiabetic potentials of PHPs are more important when compared to individual plant extracts [15]. This enhancing effect might be due to a synergetic effect between all the plant extracts when mixed together [15]. 

The previously mentioned information was the basis on which we relied to carry out this study that aims to investigate the antihyperglycemic activity of LP, one of lavender species that has never been studied for its antidiabetic activity. Moreover, its combination with PG and FGK was also studied. A special focus was given to some of its potential mechanisms of action on hyperglycemia, i.e., inhibition of activities of the digestive enzymes involved in the carbohydrate digestion (pancreatic α-amylase and intestinal α-glucosidase) and inhibition of D-glucose intestinal absorption.

## 2. Materials and Methods

### 2.1. Chemicals and Reagents

The used standards are protocatechic acid (Koch-Light Laboratories LTD, Bucks, UK), cinnamic acid (Rhône-Poulenc, Paris, France), chlorogenic acid (Sigma-Aldrich, St. Louis, MO, USA), vanillic acid (Merck, Darmstadt, Germany), gallic acid (Prolabo, Paris, France), rosmarinic acid (Extrasynthèse, Genay, France), Coumarin (Behringer, Willich, Germany), apigenin (obtained from Carl Roth, Karlsruhe, Germany), ferulic acid, and caffeic acid (purchased from Sigma, USA) as well as herniarine, luteolin, and myricetin (purchased from Sarsyntex, Merignac, France). Methanol (Carlo Erba Reagents, Val-de-Reuil, France) and formic acid (Carlo Erba Reagents^TM^, Cornaredo, Italy) were of HPLC grades. Acarbose, α-amylase and α-glucosidase were purchased from Sigma Aldrich (St. Louis, MO, USA). D(+)-glucose anhydrous, sucrose, starch and phloridzin were purchased from Sigma Aldrich (Riedel-de Haen, Seelze, Germany). Glucose oxidase-peroxidase (GOD-POD) kit was purchased from Biosystems (Barcelone, Spain). All the chemicals used were of analytical grades.

### 2.2. Plant Material

The fresh flowering tops of *Lavandula pedunculata* (Mill.) Cav. (Voucher number: RAB111854) and *Punica granatum* L. pericarp (Voucher number: RAB65559) were collected respectively from Azrou (2019) and Boulemane (2018) regions (Middle Atlas, Morocco). Identification of the plants was carried out at the Scientific Institute of Rabat (Rabat, Morocco) by Dr. Hamid Khamar where a specimen was deposited. The collected plants were then dried for thirteen days in the open air and in the shade. Aqueous dry extract powder of *Galega officinalis* L. (GO) (PR 3199) was purchased from Plantex (Saint-Michel-sur-Orge, France) and *Trigonella foenum-graecum* L. (FENU60001) from Natural functional ingredients (Saint-Sylvain-d’Anjou, France).

#### 2.2.1. Preparation of the Aqueous Extracts

Air dried flowering tops of LP or PG pericarps were mixed with distilled water (1:20; *w*/*v*) and heated for one hour at 70 to 80 °C. The mixtures were then filtered and the obtained filtrates were dried in the oven at 70 °C until obtaining the dry extract powder. The extracts were put in closed flasks away from light and humidity until further use.

#### 2.2.2. UHPLC Analysis of Aqueous Extracts

Chromatographic analysis of the aqueous extracts was carried out on an AQUITY UPLC H-Class System (Waters Corporation, Manchester M23 9LZ, UK) equipped with two independent pumps, an automatic injector, a controller, a diode array UV detector (DAD), a mass spectrometer with ESI ionization source and a quadrupole as an analyzer. The stationary phase was a reverse phase Waters^®^ Acquity BEH C18 column (2.1 × 50 mm, 1.7 µm) connected to a 0.2 µm in-line filter. The mobile phase was composed of two solvents: (A) ultrapure water (Milli-Q^®^ Integral 5, Merck^TM^, Allemagne, Germany) + 0.1% formic acid; (B) methanol + 0.1% formic acid. The elution gradient established was 0–5% B (1 min), 5–20% B (0.5 min), 20% B (3.5 min), 20–100% B (4 min), rinsing of the column 100% B (2 min) and re-equilibration 100-0% B (0.5 min), 0% B (2.5 min). Methanol 70% was required for washing the system.

The aqueous extracts were solubilized in a methanol / water mixture (1:1, *v*/*v*), so as to obtain a concentration of 1 mg/mL, then they were filtered through 0.2 μm PTFE filter. For each analysis, 4 μL of the extract were injected. The temperature was set at 30 °C and flow rate was set at 0.3 mL/min. 

This analysis was carried out on few standards chosen according to bibliographic data, and which were injected under the same conditions as those of the extracts. These standards are cinnamic acid, luteolin, apigenin, myricetin, ferulic acid, protocatechuic acid, vanillic acid, chlorogenic acid, caffeic acid, rosmarinic acid, gallic acid, herniarine, and coumarin.

Compounds were identified by matching their retention time, UV spectrum and molecular weight to those of the used standards.

### 2.3. Animals

For OGTT and toxicological studies, healthy adult *Wistar* rats (150–250 g body weight, 2–3 months of age) and Swiss *albino* mice (20–30 g body weight) were provided by the animal house of the Faculty of Science, Mohammed First University, Oujda-Morocco.

For the intestinal glucose absorption studies, 7 week old male mice (Black mice C57BL/6JRj, 20–25 g) were purchased from Janvier SASA (Route des chênes, Le Genest-st-Isle, St Berthevin, France) and were acclimated for a week in the animal house conditions of the Faculty of Pharmacy, Lille University, France (approval N° D5935010). The animals were grouped in polycarbonate cages with free access to food and water, in an environmentally controlled room (22–26 °C, ventilation, 12/12 h light/dark cycle). Experiments on animals were carried out in accordance with the internationally approved “Guide for the care and use of laboratory animals” prepared by the National Academy of Sciences and published by the National Institutes of Health [22]. All efforts were made to minimize animal suffering and the number of animals used. For OGTT studies, ethical approval was obtained from Mohammed First University, Oujda-Morocco (31/2019/LBBEH-05 and 21/011/2019).

### 2.4. Pharmacological Studies

#### 2.4.1. Inhibition Assay of Pancreatic α-Amylase Enzyme Activity

The inhibitory effect of LP aqueous extract on the enzymatic activity of pancreatic α-amylase was studied in vitro according to the method described by Bouhrim et al. (2021) with some modifications [23]. Then, 200 μL of the α-amylase enzyme solution (13 IU) and 200 μL of phosphate buffer (0.02 M; pH = 6.9) were placed in different tubes. On one hand, 200 μL of the LP extract were added at different concentrations: 2.27, 1.82, 1.36, 0.91, 0.45, and 0.23 mg/mL. On the other hand, 200 µL of acarbose were used as a positive control at several concentrations: 2.27, 0.91, 0.45, 0.23, 0.11, and 0.06 mg/mL. All the reagents were dissolved in phosphate buffer and 200 µL of this solution were used as a control. The mixtures were pre-incubated for 10 min at 37 °C before adding 200 μL of starch (1%) to each tube. The different solutions were then incubated for 20 min at 37 °C. Then, 600 μL of dinitrosalicylic acid (DNSA) color reagent (2.5%) was added to stop the enzymatic reaction. The tubes were then incubated for 8 min at 100 °C, and they were put in an ice-water bath for few minutes. At the end, the mixtures were diluted by the addition of 1 mL of distilled water and the absorbance of each tube was measured at 540 nm. The inhibition percentage was then calculated using the formula below:

(1) $$\mathrm{IP}=\frac{A_{\mathrm{control}}{\;-A}_{\mathrm{test}}}{A_{\mathrm{control}}}\;\times \;100$$

where IP: inhibition percentage (%); A_control_: absorbance of the final mixture without inhibitor; A_test_: absorbance of the final mixture in the presence of LP extract or acarbose.

The concentration of the tested substance (LP extract/acarbose) inhibiting 50% (IC_50_) of the enzymatic activity of α-amylase was determined graphically.

#### 2.4.2. Inhibition Assay of Intestinal α-Glucosidase Enzyme Activity

The inhibitory effect of LP aqueous extract on the enzymatic activity of intestinal α-glucosidase was evaluated in vitro according to the method described by Bouhrim et al. (2021) with some modifications [23]. The method consists of the quantification of D-glucose released from sucrose degradation. 10 µL of different concentrations of acarbose solutions (41, 82, 165, 328 and 656 µg/mL) or LP extract solutions (80, 170, 250, 330 and 650 µg/mL) were added to a mixture containing 100 μL of sucrose (50 mM), 100 μL of α-glucosidase enzyme solution (10 IU), and 1000 μL of phosphate buffer (50 mM; pH = 7.5). All the reagents were dissolved in phosphate buffer and 10 µL of this solution was used as a control. The mixtures were then incubated in a water bath at 37 °C for 25 min. Immediately after incubation, the different solutions were heated for 5 min at 100 °C to stop the enzymatic reaction. The D-glucose oxidase method using GOD/POD kit was used to determine the released D-glucose. The absorbance was measured at 500 nm and the inhibition percentage was calculated using the formula below:

(2) $$\mathrm{IP}=\frac{A_{\mathrm{control}}\;{-\;A}_{\mathrm{test}}}{A_{\mathrm{control}}}\;\times \;100$$

where IP: inhibition percentage (%); A_control_: absorbance of the final mixture without inhibitor; A_test_: absorbance of the final mixture in the presence of LP extract or acarbose.

The concentration of the tested substance (LP extract/acarbose) inhibiting 50% (IC_50_) of the enzymatic activity of α-glucosidase was determined graphically.

#### 2.4.3. Intestinal Glucose Absorption

D-glucose absorption through the intestinal tissue was studied ex vivo using Ussing chambers. The Black mice were left on empty stomachs with free access to drinking water for 18 h before the experiments. After killing the animals by CO_2_ inhalation, their intestines were removed and emptied of their content using a cold Ringer’s solution. Then the jejunum was cut into small fragments (1–1.5 cm) and opened along the mesenteric border. Every fragment was placed as a flat sheet between the two sides of the plexiglas Ussing chambers (exposed area: 0.3 cm^2^), allowing a mucosal compartment (luminal side) and a serosal compartment (serosal side) to be determined (blood side). Every half-chamber was filled in with 3 mL of Ringer’s solution (pH = 7.4) thermostatically controlled at 37 °C. The oxygen saturation of this solution was constantly maintained by a permanent bubbling with carbogen (95% O_2_/5% CO_2_) circulation the chambers [24].

The normal isotonic Ringer’s solution that was used contains NaCl (115 mM), NaHCO_3_ (25 mM), MgCl_2_ (1.2 mM), CaCl_2_ (1.2 mM), K_2_HPO_4_ (2.4 mM), and KH_2_PO_4_ (0.4 mM). 

The spontaneous transmural potential difference (PD) was measured using agar bridges containing KCl solution (3 M) in agar (4 percent, *w*/*v*) to represent asymmetry of electrical charges between the mucosal and serosal sides of the gut. These bridges were placed on both sides of the tissue and connected to calomel half-cells, connected to a high-impedance voltmeter. The PD was short-circuited and maintained at 0 mV by a short-circuit current (I_sc_) throughout the experiment via two stainless steel 316L working electrodes directly placed in each compartment [25], and linked to a voltage-clamp system (JFD-1V, Laboratoires TBC, France). The delivered I_sc_ (in μA/cm²), that was recorded continuously using Biodaqsoft software and corrected for fluid resistance. It represents the sum of the net ion fluxes transported across the epithelium in the absence of an electrochemical gradient (mainly Cl^−^, Na^+^ and HCO_3_^−^).

LP extract solutions were prepared extemporaneously when added to the mucosal compartment (prewarmed at 37 °C) in a volume of 250 μL, 5 min before adding the glucose (10 mM) to obtain final concentrations of 0.01 to 1000 μg/mL (glucose was replaced with mannitol at 10 mM in the serosal compartment).

Results are expressed as the difference between the I_sc_ plateau measured after 20 min of the glucose addition (without extract), and the I_sc_ plateau measured after the glucose addition preceded by the addition of LP extract. The principle is to stimulate the intestinal absorption of glucose (increase of I_sc_) in the presence and absence of LP extract at different concentrations to detect any blocking of this absorption.

#### 2.4.4. Antihyperglycemic Study in Healthy Rats

##### Acute Oral Glucose Tolerance Test

Acute OGTT was carried out using normal *Wistar* rats, left on empty stomachs for 14 h with free access to water, and which were grouped in six groups of four rats each (two males and two females). The control group (CT) was orally administered distilled water (10 mL/kg b.w.). The five test groups (LP, PG, FGK, GO, MET) were administered the aqueous extracts at 1 g/kg b.w. The rats were force-fed with the different products immediately after the measurement of their glycaemia; 30 min later, another measurement of the glycaemia was carried out, and then the rats were overloaded with D-glucose at a dose of 2 g/kg b.w. Subsequently, the variation in blood sugar was monitored every 30 min for two hours [23].

##### Chronic Oral Glucose Tolerance Test 

To assess the effect of the repeated extracts administration, a chronic OGTT was performed using the same groups of rats used above (Acute oral glucose tolerance test). The testing products were orally administered to rats once a day for four weeks (chronic treatment). After the experimental period, the rats were left on empty stomachs for 14 h, and the glycaemia measurement was carried out according to the same procedure described above (Acute oral glucose tolerance test). 

##### Acute Oral Glucose Tolerance Test for Plant Mixtures

The principle of this test is similar to the one used for individual plants which is described above (Acute oral glucose tolerance test). Four groups were studied; starting with the control group which was administered distilled water orally (10 mL/kg b.w.). Test group (LP + PG) was administered a mixture containing 50% of LP extract and 50% of PG extract at a final dose of 1 g/kg b.w. Test group (LP + FGK) was administered a mixture containing 50% of LP extract and 50% of FGK extract at a final dose of 1 g/kg b.w. MET group was administered MET (1 g/kg b.w.).

### 2.5. Toxicological Studies

#### 2.5.1. Acute Toxicity

Swiss albino mice were used to test the acute toxicity of lavender aqueous extract (LP). Mice were left on empty stomachs for 16 h before the test, and then they were randomly divided into five groups of six mice each (three males and three females). The control group was force-fed with distilled water. Test groups 1, 2, 3, 4, and 5 were administered different concentrations of LP extract (1, 3, 5, 7, and 10 g/kg b.w., respectively). The mice were given the extract only once, and then they were put under observation for 15 days. Any clinical signs and/or behavioral changes would have been considered signs of acute toxicity. 

#### 2.5.2. Subchronic Toxicity

Subchronic toxicity test was performed using the same groups of rats used for the chronic OGTT (Chronic oral glucose tolerance test). The body weights of the rats were measured weekly as they were force-fed with the plant extracts. After the experimental period, the rats were left on empty stomachs for 14 h before being sacrificed to collect their blood and organs. The biochemical parameters of the collected blood were then measured in plasma: Alanine aminotransferase (ALT), aspartate aminotransferase (AST), direct bilirubin, total bilirubin, albumin, total cholesterol, triglycerides, high-density lipoproteins (HDL-c), low-density lipoproteins (LDL-c), plasma glucose, lipase, total protein, creatinine, urea, uric acid, calcium, and phosphate. All tests were performed with the COBAS INTEGRA^®^ 400-Plus analyzer using standard clinical diagnostic kits. Also, the relative weights of the livers and the kidneys were measured. 

#### 2.5.3. Histology 

For histological examination, different tissues (stomach, small intestine, liver, spleen, and kidneys of rats) were fixed in 10% neutral formalin, embedded in paraffin, sectioned to a thickness of approximately 5 μm, stained with hematoxylin and eosin, and examined for histopathological changes under the microscope (Olympus, Tokyo, Japan).

### 2.6. Statistical Analysis

Pharmacological responses for separate experiments using *n* tissues are presented as means ± SEM (standard error of the mean). Graphs of the concentration-response curves were determined using nonlinear regression and were fitted to the Hill equation by an iterative least-squares method (GraphPad Prism version 8.0 for Windows, GraphPad Software, San Diego, CA, USA). One-way analysis of variance (ANOVA) was performed for the comparison of the different effects with the control (Dunnett) and for the multiple-group comparisons (Newman-Keuls). GraphPad Prism computed the AUC using the trapezoid rule. Statistical significance was set as *p* < 0.05.

## 3. Results

### 3.1. UHPLC Analysis

**Figure 1:**
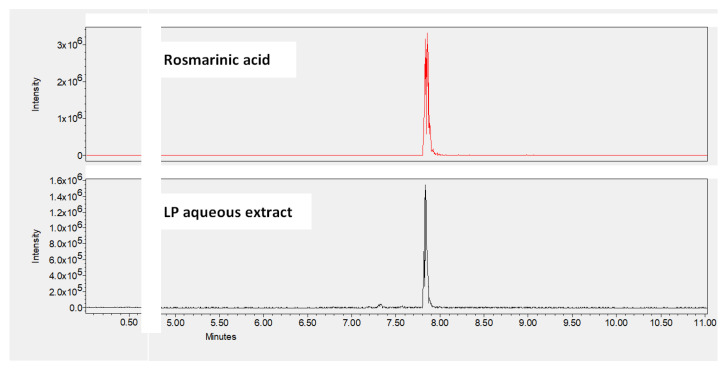
UHPLC-MS chromatograms showing the dominance of rosmarinic acid peak in LP aqueous extract.

The chemical composition of LP aqueous extract was determined using UHPLC-MS. The results (Table 1) show that the most abundant compound in this extract is rosmarinic acid. Figure 1 shows the UHPLC-MS chromatograms of rosmarinic acid detected in LP aqueous extract. Moreover, coumarin, protocatechuic acid, herniarin, caffeic acid, apigenin, luteolin, myricetin, and chlorogenic acid are present in this extract but with a lower abundance, as well as gallic acid and cinnamic acid that are present as traces. We can notice that vanillic acid and ferulic acid are absent in LP aqueous extract. 

### 3.2. Antihyperglycemic Study in Healthy Rats

#### 3.2.1. Oral Glucose Tolerance Test

To evaluate the effect of LP aqueous extract on the systemic glucose homeostasis, an OGTT was performed in conscious fasted rats after acute and chronic oral administration of this extract (LP), and we compared its effect with other plant aqueous extracts such as PG, FGK, and GO.

**Figure 2:**
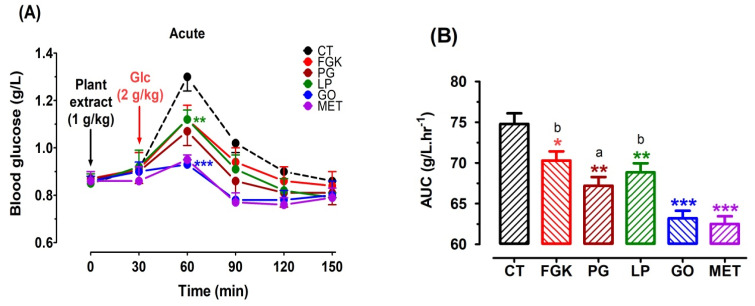

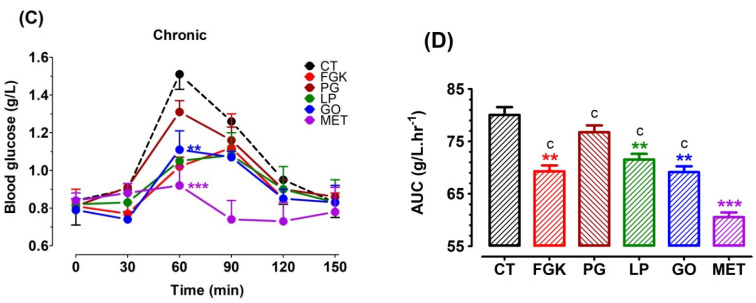
Acute and chronic OGTT after oral treatment of rats with plant extracts. Rats were force-fed intragastrically with distilled water as a negative control (CT, ■), 1 g/kg of the different aqueous plants extracts (LP, ■; PG, ■; FGK, ■; GO, ■), or 1 g/kg of the reference antidiabetic drug metformin (MET, ■) for 4 weeks. OGTT was performed on fasted rats every 30 min during 2 h after the first glucose (2 g/kg) overload (acute, panel (**A**)) and at the end of the 4-week treatment (chronic, panel (**C**)). Panels (**B**) and (**D**) show the AUCs (CT, ■; LP, ■; PG, ■; FGK, ■; GO, ■; MET, ■) for panels (**A**) and (**C**) respectively and were calculated using GraphPad Prism Version 8.0 software. * *p* < 0.05; ** *p* < 0.01; *** *p* < 0.001 in comparison with the control (Dunnett’s test). (**a**) *p* < 0.05; (**b**) *p* < 0.01; (**c**) *p* < 0.001 in comparison with MET (Newman-Keuls test).

During the OGTT, acute oral dose of LP decreased the peak glucose concentration (30 min, *p* < 0.01, Figure 2A) and the AUC (*p* < 0.01, Figure 2B). We noticed that this effect was lower when compared to that of GO and the positive control MET (*p* < 0.001), but remained close to that of PG (*p* < 0.01). 

The same findings were observed after the chronic OGTT with LP when compared to the control (*p* < 0.01, Figure 2C,D). Finally, no statistical differences between male and female animals were observed (data not illustrated).

#### 3.2.2. Oral Glucose Tolerance Test with Combinations of Plants

**Figure 3:**
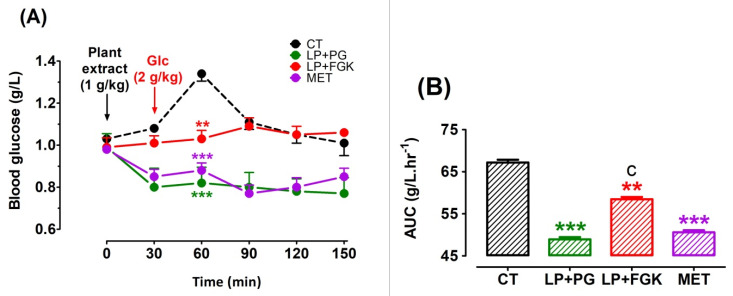
Acute OGTT after oral treatment of rats with combinations of plant extracts. Rats were force-fed intragastrically with distilled water as a negative control (CT, ■), 1 g/kg of the different combinations of the plants aqueous extracts (LP + PG, ■; LP + FGK, ■), or 1 g/kg of the reference antidiabetic drug metformin (MET, ■). OGTT was performed on fasted rats every 30 min during 2 h after the glucose (2 g/kg) overload (panel (**A**)). Panels (**B**) show the AUCs that were calculated using GraphPad Prism Version 8.0 software. ** *p* < 0.01: *** *p* < 0.001 in comparison with the control (Dunnett’s test). (**c**) *p* < 0.001 in comparison with MET (Newman-Keuls test).

To evaluate the effect of plants aqueous extracts combined with LP aqueous extract on the systemic glucose homeostasis. After acute treatment of the combination of LP + PG and LP + FGK, an OGTT was conducted in awoke fasting rats. We compared the obtained effect with the positive control (MET). Acute oral administration of LP + PG and LP + FGK reduced the peak of the glucose concentration and the AUC (*p* < 0.001 with LP + PG and *p* < 0.01 with LP + FGK, Figure 3A,B). The effect of LP + PG was similar to that of the positive control (MET).

### 3.3. Inhibition of Pancreatic α-Amylase Activity

**Figure 4:**
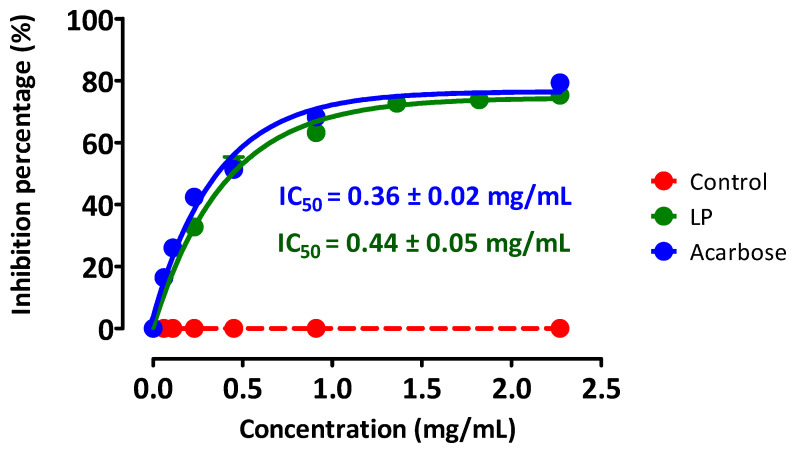
Inhibiting effect of the LP aqueous extract and acarbose on the pancreatic α-amylase enzyme activity. α-amylase was mixed and incubated with starch in the presence or absence of LP extract. Arcabose (medicine) was used as positive control.

The enzymatic activity of pancreatic α-amylase, incubated with the substrate (starch) in the presence of LP extract, was inhibited in concentration-dependent manner (Figure 4) and this inhibition was close to that observed with acarbose with IC_50_ respectively of 0.44 ± 0.05 mg/mL for LP and 0.36 ± 0.02 mg/mL for acarbose.

### 3.4. Inhibition of Intestinal α-Glucosidase Activity

**Figure 5:**
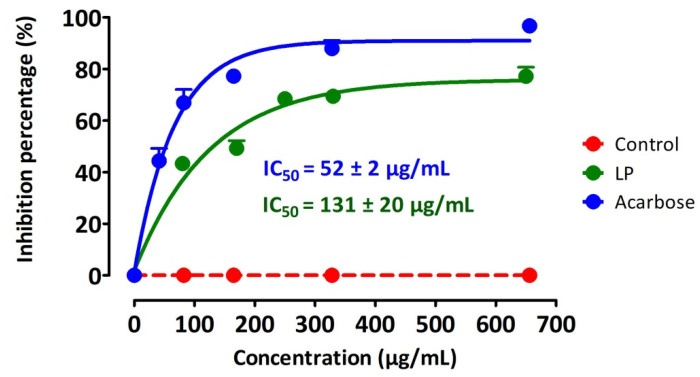
Inhibiting effect of the LP aqueous extract and acarbose on the intestinal α-glucosidase enzyme activity. Arcabose (medicine) was used as positive control.

The enzymatic activity of intestinal α-glucosidase, incubated with the substrate (sucrose) in the presence of LP extract, was inhibited in a concentration-dependent manner (Figure 5). The effect of Acarbose was more pronounced than that of LP extract. The IC_50_ was respectively of 131 ± 20 µg/mL for LP, whereas that of Acarbose was 52 ± 2 µg/mL.

### 3.5. Lavandula Pedunculata Inhibits Absorption of Sodium-Dependent D-Glucose

The impact of LP on the intestinal transit of glucose was studied using a short-circuit current method in Ussing chambers.

**Figure 6:**
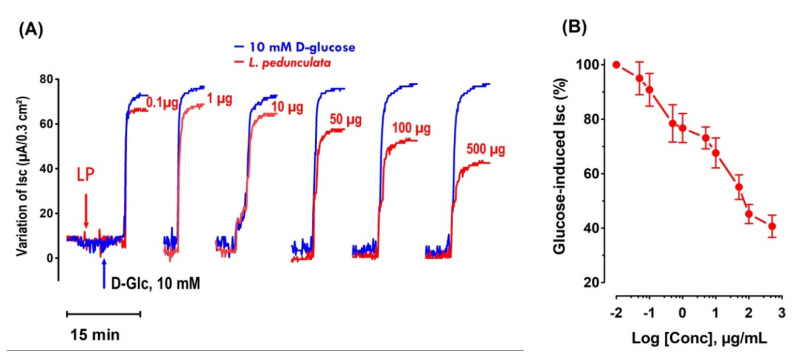
Effect of LP aqueous extract on the glucose-induced I_sc_. (**A**) Typical recording of the I_sc_ (μA/0.3 cm^2^) inhibition across mouse jejunum fragment in Ussing chambers by the addition of LP on the mucosal side 5 min before adding the D-glucose (10 mM) into the same side. This figure showed that LP induced a concentration-response inhibition of I_sc_. Increase in I_sc_ reflects a sodium-dependent glucose absorption through the sodium-glucose cotransporter-1 (SGLT1). The plateau represents the maximum increase in I_sc_. (**B**) Represents the concentration–response curve related to the use of LP extract. *n* = 6–7 tissues studied.

After a stabilization time (minimum 40 min), 10 mM D-glucose were added to the mucosal side of the mouse jejunum and the activity of the sodium-dependent glucose transporter-1 (SGLT-1) was followed as the rise in the I_sc_ sodium-dependent (Figure 6A).

The introduction of LP aqueous extract into the mucosal compartment, 5 min before adding the D-glucose (Figure 5), induced a significant and concentration-dependent inhibition of the I_sc_ (IC_50_ = 81.28 ± 4.01 µg/mL) as shown in Figure 6B. The maximum inhibition of the I_sc_ reached almost 60% of the inhibition induced by phloridzin (0.5 mM) and was obtained with a concentration of 500 µg/mL.

The inhibitory impact of LP aqueous extract on mucosal intestinal glucose absorption was not shown to be glucose concentration dependent.

### 3.6. Toxicological Studies

#### 3.6.1. Acute Toxicity 

Oral administration of the LP aqueous extract to mice at different doses (1, 3, 5, 7, and 10 g/kg b.w.) did not cause any mortality, and no signs of toxicity were noticed (diarrhea, vomiting, abnormal mobility, etc.) after a follow-up of 15 days. 

#### 3.6.2. Subchronic Toxicity 

##### Effect on Body Weights and Relative Weights of Livers and Kidneys 

**Figure 7:**
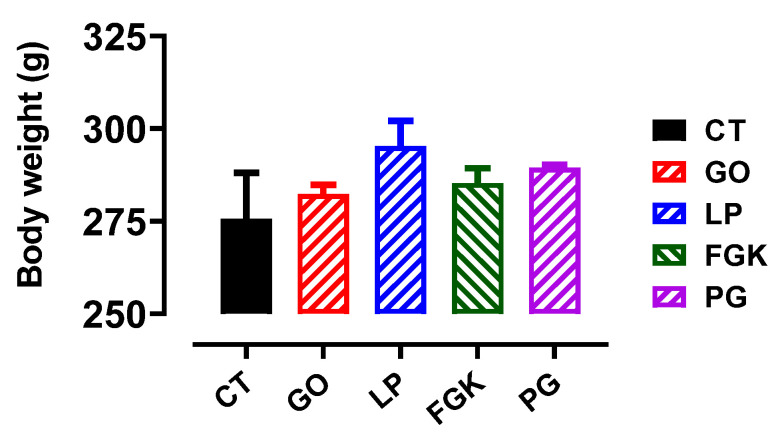
Effect of the oral administration of the plant aqueous extracts on the body weight variation in normal rats after four weeks.

The safety of the LP, FGK, PG, and GO aqueous extracts was evaluated by monitoring the variation in the body weight of normal rats during four weeks of the extract consumption at a dose of 1 g/kg b.w. (Figure 7). Also, this safety was assessed by measuring the relative weights of the livers and the kidneys at the end of the experimental period (Table 2). The obtained results showed that the variation in body weights and relative weights of organs of the treated rats is not significant in comparison with the control.

##### Effect on the Blood Biochemical Parameters

Biochemical parameters of the studied rats that were administered the LP, FGK, PG, and GO aqueous extracts for four weeks is shown in Table 3. The daily administration of these extracts at a dose of 1 g/kg b.w. did not cause any significant variation in these parameters in comparison with the control group.

#### 3.6.3. Histology

**Figure 8:**
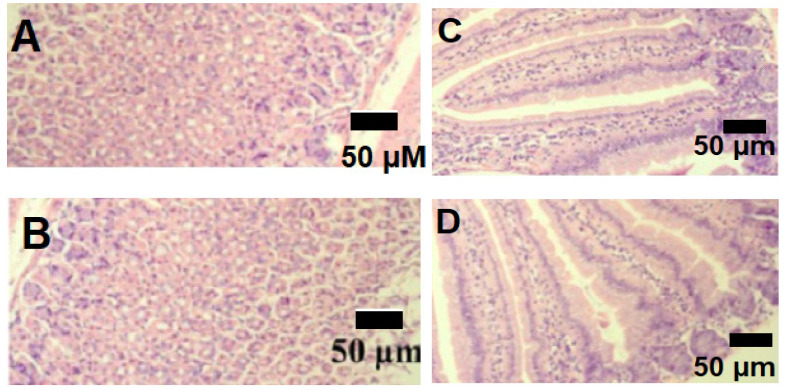
Histological analysis of rat stomach sections (**A**,**B**) and small intestine sections (**C**,**D**). The control groups (**A**,**C**) were given water and the treated groups (**B**,**D**) were given *Lavandula pedunculata* (LP) aqueous extract every day for four weeks. The figure shows the absence of any cellular modifications after the chronic administration of LP while compared to the control.

**Figure 9:**
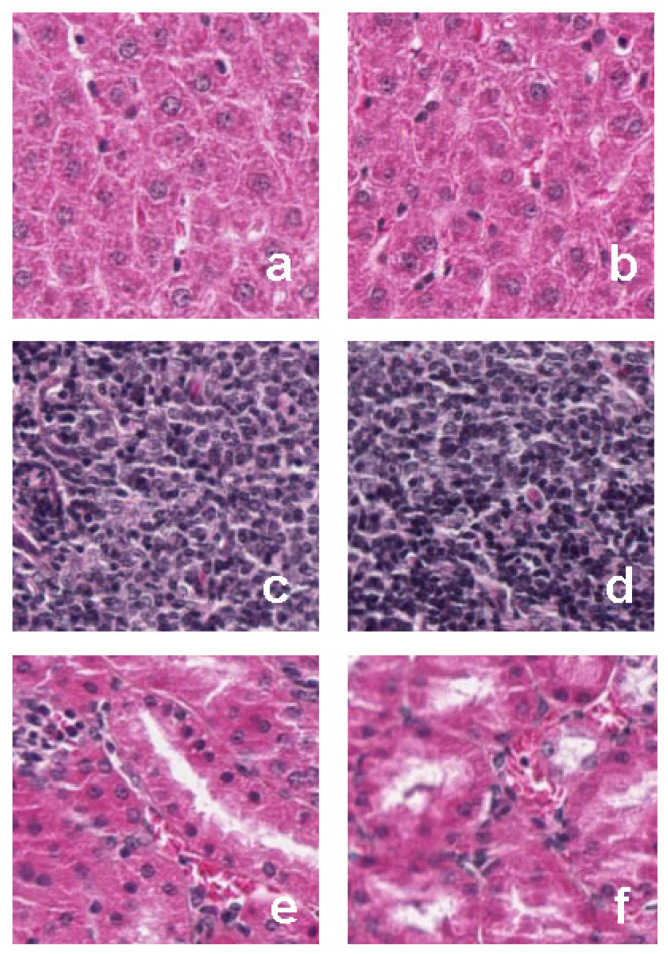
Histological analysis of tissues from control or *Lavandula pedunculata* (LP) treated rats. Rats were given LP or water every day for 4 weeks by gavage. Liver, spleen, and kidney slices were analyzed from control (**a**,**c**,**e**, respectively) or LP-treated rats (**b**,**d**,**f**, respectively). No cellular modifications were observed in the rats receiving LP in comparison with the control.

Histological analysis of rat stomach sections (Figure 8A,B) and small intestinal sections (Figure 8C,D) did not show any modifications after the chronic administration of LP (B,D) while compared to the control (A,C). Moreover, histological analysis of rat liver (Figure 9a,b), spleen (Figure 9c,d) and kidney (Figure 9e,f) slices did not show any cellular modifications after the chronic administration of LP (b,d,f) while compared to the control (a,c,e).

## 4. Discussion

UHPLC analysis of LP aqueous extract studied in this work demonstrated its high content in rosmarinic acid. Moreover, coumarin, protocatechuic acid, herniarin, caffeic acid, apigenin, luteolin, myricetin, and chlorogenic acid were also identified in this plant extract. Lopes and his collaborators identified some phenolic compounds of LP aqueous extract from different populations in Portugal using HPLC analysis. The results showed that phenolic acids are the most abundant, especially salvianolic acid B and rosmarinic acid present in large concentrations, and caffeic acid present in smaller ones. As for flavonoids, luteolin-7-O-glucuronide was the major compound in the studied extracts [26]. Another study was also conducted on different extracts of LP from Portugal that were analyzed using HPLC. The results showed high concentrations of rosmarinic acid and smaller ones of luteolin. Moreover, apigenin was present in the ethanolic and hydroethanolic extracts and absent in the aqueous ones [27]. 

Different studies demonstrated the inhibitory effects exerted by rosmarinic acid on the enzymatic activity of α-amylase and α-glucosidase. Other studies showed that this compound is able to improve insulin sensitivity and glucose uptake in animal models [28,29]. Furthermore, the antidiabetic activity of luteolin was assessed in mice and the results showed its potential in improving diabetes [30].

To our knowledge, this is the first study demonstrating that LP directly inhibits the pancreatic α-amylase and intestinal α-glucosidase enzyme activities in vitro, as well as the electrogenic intestinal glucose absorption in vitro, and that it improves the glucose tolerance in rats after acute and chronic oral administration in vivo. Although this inhibition of the intestinal glucose absorption remains low compared to that of *Arbutus unedo* root crude aqueous extract [31] and *Boscia senegalensis* seeds [32], the maximum effect is close to 60%. This work shows that LP could join the list of natural α-amylase, α-glucosidase and SGLT1 inhibitors. Acarbose, Phlorizin (a glucoside of phloretin) and other natural α-amylase, α-glucosidase, and SGLT1 inhibitors have been utilized as pharmacological agents or in the treatment of type 2 diabetes [33,34]. Moreover, LP could join the list of natural α-glucosidase inhibitors.

LP aqueous extract exhibited potent actions on hyperglycemia. It ameliorates the oral glucose tolerance in rats, and it exhibits an inhibiting effect on digestive enzymes and glucose absorption. Although there are no available studies on the antidiabetic activity of LP, there are few limited studies concerning other lavender species like *L. stoechas* and *L. officinalis*. Indeed, Bint Mustafa and her collaborators have shown in a previous study that the treatment of alloxan-induced diabetic mice with a hydroalcoholic (7:3) maceration of *L. stoechas* roots showed significant (*p* < 0.05) effects on fasting mice’s blood glucose levels in a dose-dependent manner. The results were similar to the ones obtained by the reference drug (pioglitazone; 1 mg/kg b.w. intraperitoneal). The prepared plant extract was used at different concentrations (50, 100, and 150 mg/kg b.w.). It was used for intraperitoneal injection into the experimental mice at a dose of 0.1 mL/kg b.w. [15]. The same lavender species (*L. stoechas*) was also studied by Sebai et al. (2013). These researchers used its essential oil that they had obtained by hydrodistillation of the aerial parts. After 15 consecutive days of treatment with the essential oil (50 mg/kg b.w.), the obtained chronic effect showed that *L. stoechas* essential oil treatment significantly protected against the increase of blood glucose induced by alloxan treatment of *Wistar* rats [35]. In another research, the effect of *L. officinalis* aerial parts macerated in ethanol (96%) was studied in alloxan-induced diabetic *Wistar* rats. Two doses of the extract (100 and 200 mg/kg b.w.) were used during 21 days of treatment. The results showed that the two doses of the *L. officinalis* ethanolic extract significantly (*p* ˂ 0.01) diminished the blood glucose level in comparison with the diabetic non-treated group [36].

In addition, the combination of LP with PG and FGK improved the oral glucose tolerance in rats after acute oral administration in vivo and it exhibited a higher antihyperglycemic activity in comparison with the individual plant extracts. Moreover, the activity obtained after combining LP with PG was judged to be similar to the reference drug MET. This amplifier effect obtained after combining the plant extracts could be either the result of the accumulation of their common active compounds, or a synergic reaction between their different compounds. The obtained results confirm and support the traditional use of LP in ACPP in type 2 diabetes therapy. In fact, LP is one of lavender species widely used by traditional healers in Morocco to prevent or cure diabetes in combination with other antidiabetic plants. This traditional method consists of combining plant extracts in the same homemade beverage to ameliorate or reduce the effect of healer potion. In Africa, it is one of the methods used to personalize treatment of patients, according to their own physical condition (child, elderly, pregnant woman) [21].

It was found in another study that the polyherbal preparation of *L. stoechas*, *Curcuma longa*, *Aegle marmelos,* and *Glycyrrhiza glabra* hydroalcoholic extracts at a dose of 150 mg/kg b.w. is significantly (*p* < 0.05) more antihyperglycemic than individual extracts [15]. Likewise, it was cited in an ethnobotanical study realized in Morocco that the population use a mixture of *L. stoechas*, *Artemisia herba alba*, and *Mentha rotundifolia* inflorescences in a decoction to treat diabetes [10].

ACPP is the possibility to use, in the same formulation, several plant extracts. It is also the possibility to combine/replace or alternate one or several plant extracts in the formula depending on the goal. ACPP offers phytopharmacologists a multitude of possibilities to formulate several combinations of plant extracts [32]. Polyphytotherapy offers other benefits in the fight against diabetes. It makes it possible to make a learned association of plants whose extracts act on several targets. This is perhaps the main reason why traditional healers combine and/or alternate different plants in their preparations against diabetes, because they can combine in the same preparation the extracts of plants which (1) stimulate the pancreas to release more insulin (secretagogues), (2) help the body use insulin (sensitizers), and (3) block the breakdown of starches and sugars (α-amylase and α-glucosidase inhibitors) such as LP.

For the time being, the present results are not supposed to alter the treatment of type 1 diabetes, which is based on life-saving insulinotherapy. However, it may be of interest to explore the possibility that LP, in combinative poly-phytotherapy, could decrease the amount of daily insulin requirement, as a consequence of improved oral glucose tolerance. 

This study showed that LP aqueous crude extract inhibits glucose absorption, as well as the pancreatic α-amylase and intestinal α-glucosidase enzyme activities. These two cumulative effects could justify its use as an antidiabetic by the traditional healers. In a study on the traditional use of *Arbutus unedo* as an antidiabetic in Morocco [31], various pharmacological actions attributed to natural or synthetic SGLT inhibitors were listed, which makes them a potential new class of drugs that could be used to manage type 2 diabetes, including:

1. Reducing the glycemic index: in addition to their improving effect on the glycemic control, some SGLT inhibitors, like *Boscia senegalensis* (Boscisucrophage) and *Nigella sativa* (Biodiabétine), have been shown to prevent diabetic nephropathy by lowering glycosylated hemoglobin (HbAlC) levels in experimental animals [32].
2. Intestinal SGLT1 inhibition: The impact of LP aqueous extract on glucose absorption in the intestine is concentration dependent and comparable to that of other antidiabetics [31,37].
3. Limited action on renal SGLT1: plasma glucose levels are maintained near normal thanks to SGLT1 that has the ability to reabsorb glucose in the proximal tubule, preventing thereby the fasting hypoglycemia [38].
4. Furthermore, the aim of SGLT inhibitor therapy is to lowering postprandial blood glucose levels [39].

The findings of the present study indicate that systemic glucose homeostasis is improved in OGTT in conscious fasted rats. This improvement is better when LP is used in combination with PG (Figure 1) than when LP and PG are used alone. In addition, this combination is as good as the clinical chemical drug Metformin. These results confirm the effectiveness of combining plant extracts to reduce systemic blood sugar.

## 5. Conclusions

**Figure 10:**
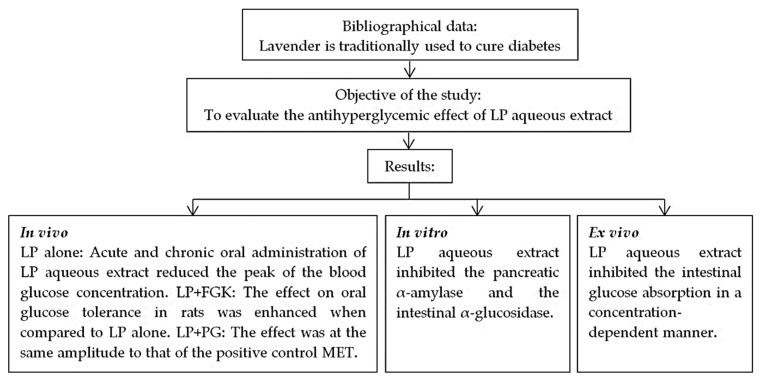
A schematic diagram of the study.

*Lavandula pedunculata* (Mill.) Cav. (LP) aqueous extract inhibited enzymatic activities of pancreatic α-amylase and intestinal α-glucosidase in vitro. In addition, this extract directly inhibited the electrogenic intestinal glucose absorption ex vivo. Moreover, LP improved the antihyperglycemic effect of PG and FGK in vivo in rats after acute oral administration (Figure 10). These findings back up the traditional use of LP in type 2 diabetes treatment and the effectiveness of the alternative and combinative poly-phytotherapy (ACPP). Still, more studies are needed to better understand all pharmacological parameters of this plant extract to be used as alternative medicine to treat hyperglycemia.

## Figures and Tables

**Table 1:** Results of UHPLC-MS analysis regarding the presence or the absence of some chemical compounds in LP aqueous extract.

Rt (min)	λ_max_ (nm)	[M − H]^−^ (m/z)	[M + H]^+^ (m/z)	Compounds	LP
6.869	278.1	WD	147	Coumarin	+
8.259	255.5, 297.2	147	149	Cinnamic acid	T
2.697	259.1, 293.6	153	155	Protocatechuic acid	+
3.923	260.3, 292.4	167	169	Vanillic acid	−
1.518	371.0	169	171	Gallic acid	T
7.761	307.9	WD	177	Herniarin	+
3.976	324.7	179	181	Caffeic acid	+
6.536	322.3	193	195	Ferulic acid	−
8.606	338.8	269	271	Apigenin	+
8.368	254.3, 350.4	285	287	Luteolin	+
7.792	253.2, 372.1	317	319	Myricetin	+
3.680	240.1, 325.8	353	355	Chlorogenic acid	+
7.831	329.4	359	WD	Rosmarinic acid	++

(WD) weak detection; (T) traces; (−) Absence; (+) presence; (++) high presence; (LP) *L. pedunculata*.

**Table 2:** Effect of the oral administration of the plant aqueous extracts on relative weights of livers and kidneys in normal rats.

Extracts	Liver	Left Kidney	Right Kidney
Control	5.08 ± 0.40	0.65 ± 0.06	0.69 ± 0.07
LP	5.83 ± 0.62	0.54 ± 0.11	0.61 ± 0.11
FGK	5.98 ± 0.52	0.59 ± 0.07	0.66 ± 0.12
PG	6.75 ± 0.72	0.57 ± 0.07	0.61 ± 0.05
GO	4.92 ± 0.61	0.53 ± 0.04	0.53 ± 0.05

**Table 3:** Analysis of the biochemical parameters of the rat’s blood.

Biochemistry	Control	LP	FGK	PG	GO
ALT (UI/L)	18.33 ± 5.86	17.33 ± 0.59	25.33 ± 4.73	20.33 ± 3.79	11.00 ± 2.65
AST (UI/L)	59.33 ± 28.38	55.67 ± 24.83	63.67 ± 16.92	59.33 ± 19.35	50.00 ± 14.53
Direct bilirubin (mg/mL)	1.00 ± 0.00	1.00 ± 0.00	1.00 ± 0.00	1.00 ± 0.00	1.00 ± 0.00
Total bilirubin (mg/mL)	1.43 ± 0.49	1.45 ± 0.49	1.27 ± 0.06	1.45 ± 0.07	1.30 ± 0.42
Albumin (g/L)	28.00 ± 2.00	25.00 ± 9.64	22.33 ± 2.08	24.67 ± 6.11	19.30 ± 6.51
Cholesterol (g/L)	0.79 ± 0.07	0.66 ± 0.25	0.86 ± 0.04	0.91 ± 0.26	0.71 ± 0.18
Triglyceride (g/L)	0.45 ± 0.12	0.40 ± 0.25	0.38 ± 0.17	0.40 ± 0.10	0.26 ± 0.04
HDL (g/L)	0.21 ± 0.04	0.23 ± 0.09	0.26 ± 0.07	0.24 ± 0.05	0.20 ±0.03
LDL (g/L)	0.11 ± 0.02	0.10 ± 0.04	0.13 ± 0.05	0.14 ± 0.04	0.10 ± 0.06
Glucose (g/L)	1.58 ± 0.30	1.93 ± 0.80	1.85 ± 0.09	1.63 ± 0.33	1.41 ± 0.62
Lipase (UI/L)	9.67 ± 3.06	7.33 ± 2.89	11.50 ± 2.12	11.00 ± 6.24	5.67 ± 2.08
Protein (g/L)	55.00 ± 16.55	44.85 ± 25.39	58.83 ± 7.26	58.23 ± 16.39	31.50 ± 7.78
Creatinine (mg/L)	6.37 ± 1.13	5.45 ± 1.95	6.19 ± 0.68	5.98 ± 1.29	4.53 ± 1.68
Urea (g/L)	0.23 ± 0.06	0.16 ± 0.06	0.23 ± 0.05	0.18 ± 0.03	0.17 ± 0.03
Uric acid (mg/L)	17.70 ± 9.72	16.33 ± 8.20	14.83 ± 2.81	23.03 ± 11.57	16.77 ± 11.72
Calcium (mg/L)	88.23 ± 15.03	84.87 ± 26.58	88.20 ± 7.62	86.80 ± 20.78	50.50 ± 5.23
Phosphate (mg/L)	52.50 ± 8.92	70.15 ± 7.71	62.65 ± 12.52	53.10 ± 18.10	59.20 ± 27.72

## Data Availability

Data are available upon request.
